# Medicinal and local food plants in the south of Alava (Basque Country, Spain)

**DOI:** 10.1016/j.jep.2015.10.022

**Published:** 2015-12-24

**Authors:** Rocίo Alarcόn, Manuel Pardo-de-Santayana, Caroline Priestley, Ramón Morales, Michael Heinrich

**Affiliations:** a Research Cluster Biodiversity and Medicines/Centre for Pharmacognosy and Phytotherapy, UCL School of Pharmacy, University of London, 29–39 Brunswick Square, London WC1N 1AX, UK; b Departamento de Biología (Botánica), Universidad Autónoma de Madrid, C/Darwin, 2, Campus de Cantoblanco, 28049 Madrid, Spain; c Lucozade Ribena Suntory Ltd., 2 Longwalk Road, Stockley Park, Uxbridge UB11 1BA, UK; d Real Jardín Botánico, Plaza de Murillo 2, 28014 Madrid, Spain

**Keywords:** F, food, H-F, Health food, I, Izki, M, medicinal, V, Valderejo, VA, Valle de Arana, VIVA area, the combined area of Izki, Valderejo and Valle de Arana., Spanish Basque Country, Food plants, Food-medicines, Traditional knowledge, Ethnopharmacology, Ethnobotany

## Abstract

**Ethnobotanical relevance:**

Medicinal and food plants in the Basque Country are an integral part of a fast changing culture. With a distinct tradition and language, this region of Europe provides an important example demonstrating the changing role of local and traditional knowledge in industrial countries. As other Mediterranean regions it preserves a rich heritage of using plants as medicine and food, offering a unique opportunity for studying the medicine food interface in an ethnopharmacological context. Therefore, the key goal of this study has been to contribute to an understanding of local and traditional plant usage, to evaluate their uses as food and medicine as well as to critically assess the role of these plants in the south of the Basque Country contributing to an understanding of how foods and medicines are used.

**Methods:**

A mixed methods approach, including participant observation; open and semi structured interviews was used. Ethnobotanical field work included 183 people, ages ranged from 24 to 98 years old with a majority being between 70 and 80 years old (mean age 71) from 31 towns of three different regions. The basic interview was a one-to-one meeting, which often included field walking and collection of samples as directed by the informants. 700 voucher specimens (most of them with duplicates) were collected for the data obtained.

Using SPSS version 20 the gathered information was processed and the replies of the different informants were subsequently organised in variables like medicine and food plants, part of the plants used, forms of preparations, zones preferred for collecting these plants. The data were analysed based on the frequency of records. This type of approach allows us to understand the way the informant’s categorize the species, and how these categories are distributed along the sample. In order to analyse the data three main categories of use were distinguished: Medicine (M), Food (F) and an intermediate Health-Food (H-F). The three categories were divided in 27 subcategories (common uses).

**Results and discussion:**

The informants recognise and use a total of 184 species from 49 families. During interviews, 5658 individual use-reports were collected relating to three use-categories – as medicines, food and health-food. The two main groups with almost the same number of species each are health-food (75 species) and (locally gathered) food only (73), with medicinal uses only (36) being the smallest group. This highlights the important overlap between food and medicines.

Overall, three core families were identified (based on the number of use reports and in the number of species): Asteraceae (25 species), Lamiaceae and Rosaceae (24 each). The most frequently reported species are *Jasonia glutinosa*, *Chamaemelum nobile, Prunus spinosa* and *Quercus ilex* subsp. *ballota.*

The most important general use-subcategories are as raw vegetables (27.43% of the use-reports and including 81 species), infusions (14.74%/42) and gastrointestinal (12.53%/42). Conceptually foods and medicines are clearly distinguished but the intermediate group of health foods is more ambiguous.

**Conclusion:**

Food and medicinal uses of plants are culturally closely linked. A wide range of plants are known and many still used. The analysis shows that the Basques use a wide range of species which are typical for Western European cultures. In comparison to other studies in the Mediterranean countries there are many similarities in the uses of different families, species of plants and their use and preparations. Some of these plants are key Mediterranean species, often used for a multitude of uses as food and medicine.

## Graphical abstract

**Figure:**
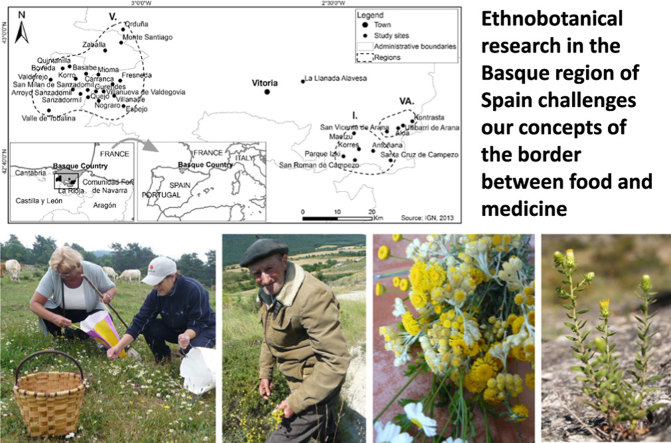

## 1 Introduction

“*… to draw the attention of ethnopharmacologists to the dietary dimension of plant utilisation. The conventional approach in ethnopharmacology is to focus on the medicinal properties of plants without adequately exploring other categories of use. As a result, we are unduly circumscribed in our understanding of the extra-nutritive aspects of food phytochemistry*’’ (Etkin and Ross, 1991, p. 25).

Nearly a quarter of a century has passed since Nina Etkin and Paul J. Ross led the ethnopharmacological discussion towards a (renewed) emphasis on the interface of food and medicine. It has been argued convincingly that we should set aside a place for food and try to understand its health promoting effects (Pieroni and Price, 2006).

Health(y) food has also entered mainstream discussions in many countries and the idea of “let food be your medicine” (attributed to Hippocrates, 460-377 BCA) is again a popular concept. An important aspect that is being highlighted in this discussion is the importance of today’s interest, from the markets and people, in functional foods, tailor-made to suit specific groups (e.g. the elderly, the young, physically active people, and people with specific conditions). The quest for diets which allow for a healthy ageing is strongly linked with increased life expectancy and larger financial assets of some sectors of the population (e.g. Heinrich and Prieto-García, 2008; Keatinge et al., 2010).

Indeed one of the well-known benefits of the Mediterranean diet is its long life expectancy (Willett et al., 1995) and many ethnobotanical surveys around the region show the importance of wild food plants and specifically wild vegetables in the Mediterranean diet (e.g., Pieroni et al., 2005; Rivera et al., 2005; Tardío et al., 2006; Leonti, 2012). Several authors have highlighted that one of the reasons that explain the prevalence of its consumption is that they have a clear positive influence on health (Leonti et al., 2006, Sánchez-Mata et al., 2012). For instance, a review of the wild vegetables traditionally used in Spain showed that 23 % of them were also orally taken as medicinal plants. They were mainly used to prepare infusions drunk to cure many different complaints, but some species were eaten with a specific medicinal purpose (Tardío, 2010). *Urtica dioica* leaves are for instance taken in omelettes against hoarseness (Pardo-de-Santayana, 2008) or hypertension (Bonet and Vallès, 2002). In the case of Southern Spain, a study made in Sierra de Alcaraz and Serranía de Cuenca showed an even higher rate, since 58% of wild foods had also medicinal uses (Rivera et al., 2005).

Besides its role for curing, they also play a major role as preventive remedies. People for instance consume many herbal teas such as *Chamaemelum nobile* L. or *Mentha pulegium* L. after meal to prevent indigestion (Pardo-de-Santayana et al., 2005, Menendez-Baceta et al., 2014). Other examples of valuable species are olive, garlic and lemon (Rivera and Obón, 1993).

Besides the local perception of the health benefits of wild foods, the high nutritional interest of many of these species is also well-known (e.g. Flyman and Afolayan, 2006; Guarrera and Savo, 2013; Morales et al., 2013). Many contain high amounts of vitamins and other antioxidants such as flavonoids, carotenoids of polyphenols, minerals, fibres and essentials fatty acids, commonly in higher amounts than their cultivated relatives (Tardío, 2011). For instance, Zeghichi et al. (2003) found high antioxidant and mineral levels in the 25 Cretan species studied. The phenolic content was remarkably high in *Crepis vesicaria* L. Another interesting species is *Montia fontana* L., with appreciable amounts of vitamin C, Mn, and very high lipid content being one of the richest source of omega-3 fatty acids among leafy vegetables (Tardío et al., 2006). The young shoots of *Asparagus acutifolius* L., *Humulus lupulus* L., *Bryonia dioica* Jacq. and *Tamus communis* L. are richer sources of carotenoids than many of the commercially available leafy vegetables (García‐Herrera et al., 2014).

Therefore, local pharmacopoeias and gastronomies of Mediterranean industrialised countries have received growing attention (e.g. Pieroni et al., 2002, 2004; Hanlidou et al., 2004; Scherrer et al., 2005; Maxia et al., 2007; Hadjichambis et al., 2008; Nebel and Heinrich, 2009; Novais et al., 2004; Pardo-de-Santayana and Macía, 2015) and this has been linked to the impact of written traditions which have facilitated the dissemination and continued use of these medicinal plants (Leonti, 2011). Spain being one of the regions where more such ethnobotanical studies have been conducted because of its high biological and cultural diversity (e.g. Rivera et al., 2005, 2006; Tardío et al., 2005; Pardo-de-Santayana et al., 2007; Parada et al., 2009; Rigat et al., 2009; Benítez et al., 2010; González et al., 2010, González et al., 2011a, González et al., 2011b; Carrió and Vallès, 2012; Viteri Alarcόn, 2012; Rigat et al., 2013).

There is therefore relatively abundant information on inventories of medicinal and wild food taxa in Spain, but there are still poorly studied regions such as the Basque Country Autonomous Community (also known as Euskadi). Euskadi is one of the regions of the Basque Country (also called Basque Country greater region in order to differentiate it from the Basque Country Autonomous Community). The Basque Country comprises territories in northeastern Spain and southwestern France with a total population of 2,900,000 inhabitants (Barandiaran and Manterola, 2004). Its geographical position, at the interface of the Mediterranean and Eurosiberian botanical regions, offers a variety of climates and a diversity of ecosystems with a resulting high level of biological diversity.

Moreover Euskadi cultural diversity is very high, with two languages (Basque or Euskara) spoken within only 7234 km^2^. Given its ethnic and cultural singularities, it has a long tradition of ethnographic studies e.g. (Barandiaran and Manterola, 1990, Barandiaran and Manterola, 2004) but its medicinal and food plants have been only recently addressed with an ethnobotanical perspective (Menendez-Baceta, 2015, Menendez-Baceta et al., 2012, Menendez-Baceta et al., 2014, Menendez-Baceta et al., 2015). In the last years several studies have been conducted in the adjacent regions Navarre and Cantabria: including Akerreta et al., 2007a, Akerreta et al., 2007b, Akerreta et al., 2010, Calvo et al., 2011, Calvo et al., 2013, Calvo and Cavero, 2014, Calvo and Cavero, 2015), Cavero et al., 2011a, Cavero et al., 2011b and Cavero and Calvo, 2014, Cavero and Calvo, 2015), on Navarre; Pardo-de-Santayana (2004), on Cantabria. None of these studies provides comparative information on the use of food and medicinal species with the exception of a study carried out in the south of Biscay (Menendez-Baceta et al., 2012). The paper states that wild foods have not a clearly perceived medicinal role in the region. An exception was the use of mints for seasoning milk since this flavoured milk was recommended against intestinal worms. On the other side herbal teas were mainly perceived as medicines, and only few people drunk *Chamaemelum nobile* tea besides its medicinal function. The use of herbal teas in food contexts is locally not considered traditional and as Basque people are a pre-Indo-European ethnic group with marked differences with the surrounding regions they are not very permeable to such kind of new customs (Sõukand et al., 2013). Maybe one of the costumes of chewing leaves might be a missing link in the food-medicine continuum (Menendez-Baceta et al., 2012).

Euskara was spoken throughout the Basque territories until the Middle Ages, but today the predominant languages are Spanish and French. Basque is a language with no known linguistic relatives, spoken by about 660,000 people mainly in the north of Spain and the southwest of France. For centuries there was no standard orthography, and Euskara was written with Romance spelling conventions (Baztarrika et al., 2010). In 2006, Euskara was the main language for 25% of the population of the Basque Country, most of them living in Biscay, Guipuzcoa and northern Navarra (Baztarrika et al., 2010). In the rural areas of Alava around Vitoria where these study was carried out the main language is Spanish.

AIMS: Given the lack of ethnobotanical research on the interface food and medicine and using the Basque Country as an example, we wanted to investigate the question: *How do the* inhabitants *in three regions* *of Alava in the Basque Country use the local flora especially as it relates to their use as food and medicine?* this study focuses on the use and knowledge of food and medicinal plants of Alava. The specific aims of this work were: (1) to describe the domain of medicinal and wild food plants, (2) to assess the cultural importance of the different species and food and medicinal categories and (3) to compare with other Mediterranean regions and see if the plant species and uses were similar.

## 2 Geographical and cultural background

### 2.1 Region of study

**Fig. 1:**
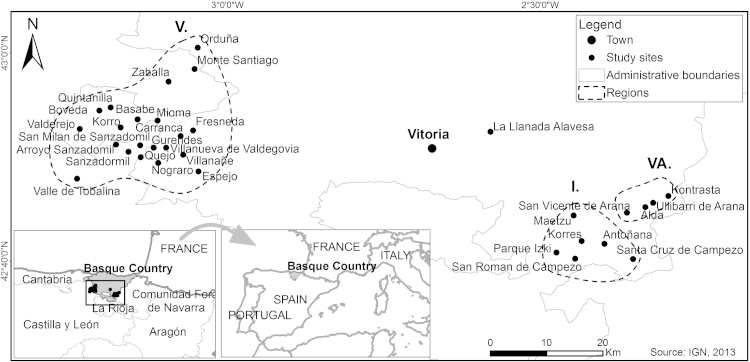
Region of study - The Alava Region in the Basque Country, Northern Spain (modified after IGN 2013).

Research was conducted in communities of regions with high levels of biodiversity where forest and undisturbed areas remain. We selected three mountainous regions of the south of Euskadi in the province of Alava: Valdegovía, Valle de Arana and Izki (Fig. 1). They belong to two different geographical regions, Valdegovía to the *Valles Alaveses* (Alavan valleys) and Valle de Arana and Izki to the *Montaña Alavesa* (Alavan mountains). They are situated on the transition between the Mediterranean and Eurosiberian biogeographical regions. The main forest communities are dominated by oaks (*Quercus pyrenaica, Q. faginea* and *Q. ilex*), beeches (*Fagus sylvatica*), pines (*Pinus sylvestris*) and boxes (*Buxus sempervirens* L.), Izki including the largest reserve in Europe of *Quercus pyrenaica* forests (Marañón and Quintana, 1993). Two nature reserves are included in the study area, Valderejo in Valdegovía and Izki, which gives the name to one of the regions.

Until a few decades ago the economy was based in an extensive and diversified agriculture, but have now changed to a more intensive and specialised crop production. For example, the region of Valle de Arana is well known for its potato plantations (Martínez Fuertes and Arriola Loiola, 2003). As happened in other Spanish and Portuguese regions, these changes in the way of life of rural people have severely eroded knowledge and customs related to the exploitation and management of most wild resources (Pardo-de-Santayana et al., 2007). From 1940 to 1960 pharmacies spread throughout the Basque Country, resulting in a loss of medical traditional knowledge (Barandiaran and Manterola, 2004).

The last decades have seen a development and promotion of tourism, due to the presence of natural parks which offer a rich biodiversity and an aesthetically pleasing landscape for the tourists. As well as natural beauty, both regions offer cultural activities, such as hunting, collecting mushrooms or visiting museum and villages.

The areas share similar demographics as all have an aged population. The density of population is low with the exception of Maeztu and Campezo (Izki), where density is higher due to the presence of industry. There is therefore a small proportion of people that have remained in their rural environment, exemplifying the last people depending on their environment and local resources. Generally, these small communities are surrounded by patches of fields where one can obtain some of the wild products used to prepare traditional medicines, foods or beverages. Today, most of these traditional activities have diminished, but some young people still practise shepherding, milking and gathering wild products, something that people consider part of their Basque culture (Menendez-Baceta et al., 2012).

Basque was spoken until the eighteenth century in most of the regions, but today Spanish is spoken in all three regions (Martínez de Madina and González de Viñaspre, 2012). For the present study these regions will be called Valdegovía, Izki and Valle de Arana, collectively as VIVA using the initials of the areas (V-I-VA).

### 2.2 Valdegovía (in *Valles Alaveses*)

Valdegovía has an area of 238.5 km^2^ and its average altitude is 552 m above the sea level. There is a population of 1148 inhabitants scattered in 24 different villages and hamlets, most of them with only 30 or 40 occupants due to migration. The area's inhabitants are highly dependent on agriculture as a source of main income. The main crops are wheat, barley, oats, rye, various other grains, potatoes, apples, cherries, pears, legumes and various vegetables. The area also has a vast amount of livestock, poultry and horses. Aside from agriculture the area receives some income as a result of touristic activities related to hunting (González, 2003). The Natural Park of Valderejo which opened in 1992 has become a very important part of the area’s economy and life. Given the strong personal, historical and cultural links of Valdegovía with the people of the surrounding villages of the province of Burgos (see Fig. 1), seven villages from Burgos were also included in Valdegovía.

### 2.3 Izki and Valle de Arana (in *Montaña Alavesa*)

This region is made up of six municipalities (Arraya-Maeztu, Bernedo, Campezo, Valle de Arana, Lagrán and Peñacerrada-Urizaharra) covering an area of 534 km^2^. Four of these six municipalities were selected for the study: Arraia-Maeztu, Bernedo, and Campezo forming the Izki area and Valle de Arana. The population (3181 inhabitants) is concentrated in small villages which are in close proximity to each other, only Maeztu and Santa Cruz de Campezo having more than 250 inhabitants while in the rest 30 out of the 46 have less than 50 inhabitants. In this region people tend to be specialized in agricultural activities and the majority of the population are pensioners. The construction and services sector is only important in Campezo and Maeztu. There are very few large businesses and a moderate number of micro-enterprises. Some specialise in forestry and others in producing asphalt and chemicals. Rural tourism is increasing its importance, especially because of people visiting the Izki Natural Park (PRO-Izki, 2015). Many people commute every day for work in Vitoria, the main city and capital of Alava.

## 3 Methods

### 3.1 Ethnobotanical data collection

Ethnobotanical fieldwork included 183 consented interviews (75 male and 108 female) conducted between November 2006 and November 2009. Informant age ranged from 24 to 98 years old with a majority being between 70 and 80 years old (mean age 71). They were selected using a snowball sampling technique since emphasis was made in selecting expert informants (Espinosa et al., 2012). We visited the retirement's homes were elder people spend the day and schools were children and teachers provided names of their relatives who know about plants and uses.

The main goal of the interviews was to understand traditional food and medicinal uses practiced in the area and how these categories overlap. The basic interview was a one-to-one meeting. The interviews were conducted in Spanish and had two main parts:

• A fixed structured part were the same questions were always asked focusing on: (a) local socio-economic environment, (b) plants and plant parts used in the past and nowadays as medicines and/or food; how were they prepared, (c) local plant names, and (d) habitat where each species grow and places of collection.
• A semi-structured free and fluent conversation, where the participants were encouraged to explore tangent aspects and details which often reveal very useful information about the area that provides precision and reliability to the information.

Interviews often included field walks and collection of samples as directed by the informants, depending on the physical condition and time availability of the participant. Walks through allotments, gardens, managed woodlands, farms, grasslands, marshlands and cliff faces were essential for providing botanical samples and identification. During these walks first-hand knowledge could be obtained from watching the participant interact with their surroundings. Open ended questions arose from watching their actions.

In most of the cases after the first interview, other meetings were agreed for providing deeper information on the plants collected and their food and medicinal uses. A collection of plants (ambulant herbarium, medicinal plant samples) was made to show to the informants. Books and photos of plants and landscapes where also used to help informants remember locations where the plant grew and to help in the identification of the species. Finally, practical activities such as cooking the dishes or preparing the medicinal plants were also organised.

The vouchers collected during the walks were dried and preserved in the herbarium of the Centre for Pharmacognosy and Phytotherapy, UCL School of Pharmacy following standard botanical techniques (Martin, 1995, Alexiades, 1996) and mostly also deposited at the Museo de Ciencias Naturales de Álava (VIT). Identification is largely based on Aizpuru et al. (2003), García López and Allue Camacho (2004), as well as López de Guereñu (1975). For botanical nomenclature we follow *Flora iberica* (Castroviejo et al., 1986–2014) for families included therein, and Flora Europaea (Tutin et al., 1964–1980) for the rest.

### 3.2 Data analysis

The data collected in the field were organised in a database. Information was structured in use-reports (UR) (Ankli et al., 1999). URs are commonly defined as the informant **i,** mentions the use of the species **s** in the use-group **u**, (Tardío and Pardo-de-Santayana, 2008). As we found important differences within the regions of study on the plant parts used and the preparation and administration methods we included both aspects in our definition of a UR. Therefore in our study a UR was defined as the event in which informant **i,** mentions the use of the plant part **p** of the species **s** prepared and administered with the method **m** in the use-group **u**.

Three main use-categories were considered: Medicine (**M**), Food (**F**) and Health-Food (**H-F**):

• The group of medicinal plants (M) contains those species that were used in the area only for medicinal proposes, to prevent, heal and recover from different health conditions.
• Food plants class (F) includes the species that were ingested daily, but were not reported to be used medicinally in the area.
• The category of Health-Food plants (H-F) includes the species that have food and medicinal uses in the area.

The three categories were further divided in 27 subcategories each that account for the local concepts, views and experiences. For instance, burns, furuncles, acne, warts, herpes, wounds, skin ulcers were grouped in the medicinal group dermatological disorders.

The quantitative analysis of uses is based on species with three or more URs and we have only focused on data, where a specific use has been reported, i.e. generic responses like “as a tonic”, “food use” were not considered.

Data collected were compared with unpublished (Puentes Amestoy, c. 1960) and published information from Basque ethnobotanical studies (Menendez-Baceta et al., 2012, Cavero et al., 2011a, Cavero et al., 2011b, Calvo et al., 2011), other Iberian works (Tardío et al., 2006, Pardo-de-Santayana, 2004, Rivera et al., 2006) and from Italy (Nebel, 2005, Nebel et al., 2006, Nebel and Heinrich, 2009, Lentini and Venza, 2007).

## 4 Results and discussion

### 4.1 Food, health foods and medicines

Table 1 presents the top 25 species according to their number of UR and the rest of the species with three or more URs are included in Appendix 1. Species with less than three URs were not included in the Appendix, since it is commonly accepted that they are less reliable (Johns et al., 1990, Le Grand and Wondergem, 1987). Overall, 184 species of 49 families have been recorded in the different regions of study based on a total of 5658 individual URs. The vast majority of all species are used for food purposes, i.e. exclusively as a food or as a health food, respectively (food, F: 73 species; health food, H-F: 75) while medicinal uses (M) account only for 36 species.

Almost 40% of the all species are in the complex group of health foods. These species are in essence food plants with a locally acclaimed health claim. This highlights the important overlap between food and medicines (Etkin and Ross, 1982). Scientifically they have often been classified as either a medicine or a health food, but the interconnectivities are complex and in these cases we classified these species as H-F. These resources may have different roles or functions in the study area:

1. Most plant uses are classified in essence by function, i.e. a certain preparation is used as a health food and as such as part of the regular diet or it is used for a specific medicinal purpose (difference by function). For instance, *Juglans regia* fruits are usually consumed raw before lunch or as an afternoon snack without any conscious medicinal use, but are also consumed specifically to prevent high cholesterol and heart problems.
2. In other cases the function is different in terms of what plant parts are used, the fruit may be a healthy snack and the leaves a medicine with a specific role (difference based on the botanical drug used). For instance, *Sambucus nigra* berries are eaten raw or used to prepare jams, while the infusion of the flowers is taken for colds, and its branches are burnt for inhalations against colds.
3. In some cases the difference is based on the form of preparation of the product to be consumed (difference based on the form of preparation). For instance, *Hypericum perforatum* flowering shoots are used to prepare a herbal tea, and also to prepare an ointment called *pomada sanjuanera*, that is prepared frying them together with many other plants.

A very large share (39.66%) of all species used belong to three families: Asteraceae (25 species), Lamiaceae and Rosaceae (24 each). This is similar to other studies in the Mediterranean (Pieroni et al., 2002, Novais et al., 2004, Pieroni et al., 2004, Guarrera et al., 2005, Rivera et al., 2005, Scherrer et al., 2005, Leonti et al., 2006; Nebel, 2005; Rivera et al., 2006; González-Tejero et al., 2007; Maxia et al,. 2007; Guarrera et al., 2008; Parada et al., 2009; Calvo et al., 2011) and in other parts of the world such as North America (Moerman et al., 1999). Menendez-Baceta et al. (2014) also reiterate the importance of Asteraceae and Rosaceae in the local pharmacopoeia of other Basque regions in Alava and Biscay. Menendez-Baceta et al. (2012) reported on the importance of the Rosaceae and Fagaceae in human food, too.

### 4.2 Plants used as food and medicine

Plants in this group have a double usage or one use which is clearly at the food-medicine interface and include foods and beverages with specific acclaimed health benefits. In essence this functionalistic distinction is one which is linked to the perceived (i.e. emic) benefits, but at the same time this group also is somewhat artificial, since it is not recognised by people as a distinct group. Also included are species which have dual uses, both more in a food context and one which is medicine-centred.

Most of the top 25 species according to their number of UR are H-F. *Jasonia glutinosa* (6.6% of the UR)*, Chamaemelum nobile* (6.2%), *Prunus spinosa* (4.7%), *Quercus ilex* subsp. *ballota* (4.3%), *Santolina chamaecyparissus* (3.7%) and *Thymus vulgaris* (3.0%) stand out as being central to the inhabitants of the region (Table 1). Seven species are food only (*Castanea sativa, Rubus castroviejoi, Rubus caesius, Fagus sylvatica, Arbutus unedo, Corylus* and *Fragaria vesca*). These species are widely used and have a high cultural salience.

This calls into question an important paradigm in ethnopharmacology, and we need to consider ways to present medicinal and food properties in an integrated way (cf. Etkin and Ross, 1991; Rivera et al., 2005). Our informants generally do not draw a very strict line between food and medicinal plants, highlighting the ambivalent nature of these two categories. The majority of the informants recognise that food plants can prevent or heal disease or “cleanse” the body.

Plants are used according to the needs of the people and different preparations, uses, applications, etc. Culturally speaking, people will manage the plants with proactively focusing on their uses for ailments, diseases, as food, beverages, etc., with the intention of preventing health problems from emerging, or subsequent treatment, if they do emerge.

The challenges of the borderline between food and medicines is well illustrated by *Jasonia glutinosa* (*té de roca*) and *Chamaemelum nobile* (*manzanilla*) which are common and important social beverages reported in the VIVA regions and generally prepared as infusions or macerations. An infusion of *J. glutinosa*, is the most popular local tea and in many Spanish regions is widely available as a speciality beverage, also served in restaurants (Pardo-de-Santayana and Morales, 2004, Pardo-de-Santayana et al., 2005). Consequently, its importance in local Basque phytotherapy and as a health food is not surprising. It is used both as an herbal tea for general use and specifically to help with digestion, in case of stomach pain or diarrhoea. There clearly is an overlap between these uses and there can be no sharp dividing line between a general use just as a food (i.e. without any health-related expectations), as a health food or as a specific medication to treat stomach pain, or diarrhoea (i.e. they are *medicinal by function*). Both its use as a general herbal tea and as an herbal medicine have a high number of use reports. Its popularity is linked to the chemical profile of the species and especially the high content of essential oil rich in camphor, borneol and *cis*-nerolidol (Pardo-de-Santayana et al., 2005) and one can link these constituents both to its use as a food and as a medicine.

The situation is very similar in the case of *Chamaemelum nobile*, the second most popular species again with uses as a medicine in case of gastrointestinal disorders, as a general digestive or as a herbal tea with a more general usage. In another region in the Basque Country, *C. nobile* is the most commonly used species used for stomach-aches and digestive pains and disorders (Menendez-Baceta et al., 2014). The uses and names of the species have remained important in the area at least since 500 years ago as mentioned by Puente Amestoy (c. 1960) in his study of the inedited manuscript *Libro de plantas* (Plants book) of Fray Juan de Vitoria written in 1587. These two species, along with *Santolina chamaecyparissus* (1.5%) and *Anthemis arvensis* (2.0%) stand as an example of aromatic plants important in local Basque culture as herbal teas and digestive infusions. The three species are called *manzanilla* (chamomile), a generic term that has been used in Spain to refer to many species used for treating digestive conditions (Pardo-de-Santayana and Morales, 2006). Many other species including *Lithospermum officinale, Artemisia alba* and *Jasonia tuberosa* are used to prepare non-alcoholic beverages, mainly herbal teas.

The third most cited species is *Prunus spinosa* which fruits are mainly used for preparing a very popular liqueur called *patxaran* or *pacharán*. Many of its uses were also mentioned in the manuscript of Juan de Vitoria (Puente Amestoy, c. 1960). Liqueurs, like herbal teas are important social beverages. They are generally prepared by combining several herbs, fruits and nuts with 17 species being used (3.98%). The most quoted were the fruits of *Prunus spinosa, Prunus avium, Malus sylvestris, Rubus ulmifolius, Cydonia oblonga* (all Rosaceae) and of *Arbutus unedo, Quercus ilex* subsp. *ballota*, *Juniperus communis, Juniperus oxycedrus* and *Juglans regia*. Important herbs were *Chamaemelum nobile*, *Helichrysum italicum*, or *Berberis vulgaris*. These liqueurs again are health foods, since they are drunk while socializing after meals, but also with the expectation of being a digestive, and they are used to manage minor gastrointestinal complaints.

Another key group of products are the various fruits processed in a variety of ways. The forth most cited species is *Quercus ilex*. subsp. *ballota*, widely used as a snack (toasted fruit) and as a coffee substitute. In this case explicit medicinal uses are much rarer and are specifically for treating herpes. Many other species may be used as ‘coffee substitutes’ including *Hordeum vulgare* (0.6% of UR), *Castanea sativa* (0.4%), *Quercus ilex* subsp. *ilex* (0.3%), *Cichorium intybus* (0.2%), *Quercus faginea x Quercus pyrenaica* (0.2%), *Quercus faginea*, *Quercus* sp., *Vicia faba* (0.1%). The choice is generally based on flavour of the processed fruit or seed which can vary between sweet, bitter and astringent. Combinations and variations of the flavour make the beverages unique and special. For example, the seeds of *Castanea sativa* are toasted then ground and boiled or infused in hot water. The roasted roots of *Cichorium intybus* are widely used combined with different species of *Quercus.* During and after the Spanish civil war *Cichorium intybus* was an important coffee substitute and its use is still remembered throughout Spain. (Guzmán, 1997, Triano et al., 1998, Fernández Ocaña, 2000, Bonet and Vallés, 2002, Tardío et al., 2005). Other species, such as *Vicia faba* are used in a similar way to the Ecuadorian Highlands (runner-bean coffee or *café de haba*).

Condiments are also represented in the top 25 species. *Thymus vulgaris* is the sixth most cited species, being mainly used for treating respiratory disorders and for seasoning meat and olives. *Origanum vulgare* and *Rosmarinus officinalis* are also highly valued. People in the VIVA region have a wider preference for plants used for seasonings than in other Basque regions where people do not consider the use of spices as a Basque tradition since they associate condiments with immigrant populations from central and southern Spain (Menendez-Baceta et al., 2012, Menendez-Baceta, 2015).

A large number of species have a use as a general “tonic” or because they are simply considered to be healthy. However, this group is not discussed further and the following analysis concentrates on preparations with specific health claims.

Most key species (*Prunus spinosa, Quercus ilex* subsp*. ballota*, *Santolina chamaecyparissus*, *Thymus vulgaris, Sambucus nigra, Juglans regia* or *Rubus ulmifolius)* are also important food and/or medicinal plants in many other Spanish regions (Leonti et al., 2006, Tardío et al., 2006, Quave et al., 2012). Obviously the similitude is higher with the neighbouring regions. The highest similitude was found with Middle Navarra, an area that borders VIVA in Valle de Arana (Cavero et al., 2011b). Four of the top 5 species are shared (*S. chamaecyparissus*, *Jasonia glutinosa*, *Chamaemelum nobile*, and *Prunus spinosa*). Other Navarran regions are not so similar. For instance in Middle Navarra only three of its top 5 medicinal species (*S. chamaecyparissus*, *Thymus vulgaris*, and *Rosmarinus officinalis*) are among the top 25 of VIVA (Calvo et al., 2011), and only two in Northern Navarra (*C. nobile*, and *Tanacetum parthenium*) (Cavero et al., 2011a). The similitude with the wild food plants consumed in other areas of Euskadi (southern Biscay and northern Alava) is also quite high, since four of the top 5 wild food species (*P. spinosa, R. ulmifolius, Castanea sativa* and *Fagus sylvatica)* are in the TOP 25 of VIVA. More differences appear when the medicinal plants of southern Biscay and northern Alava are compared. None of the top 5 medicinal species are in the top 25 of VIVA (Menendez-Baceta et al., 2014). Nearly half of the wild food plants mentioned in Gorbeialdea, are shared with VIVA area (Menendez-Baceta et al., 2012).

Such similarities may either be based on an exchange of practise or on parallel developments. Keeping the interconnected histories of the people in Northern Spain in mind, an exchange of practice seems to be the most plausible explanation. A standardisation of the knowledge is based on historical events that can strengthen or weaken the knowledge of plants, according to the needs of the people for a given time period. For example, Weckerle et al. (2009) argues that under Mao's government important information and books were distributed in order to improve the health care system. Herbals have exerted a strong influence increasing the similarity of plant knowledge among rural populations in China. However, distinct local use of plants also exists, indicating that plant knowledge, specific to each rural community, is alive and practised. In the Basque Country, after the civil war in 1936, much of the traditional knowledge of species was lost (Barandiaran and Manterola, 2004). This is an indicator that political currents have a strong influence on whether people preserve their knowledge, or whether the knowledge is lost (Menendez-Baceta et al., 2015).

Overall, this study highlights that due to centuries of interconnectivities, there is a strong overlap in practices and there are many instances of shared practice, pointing both the usefulness of these species and to the active sharing of knowledge and practice. Interestingly, the similarity of the area with the rest of the Iberian Peninsula is higher than in Basque areas where Basque is still the main language (Menendez-Baceta et al., 2015).

### 4.3 Medicinal plants: Identifying key species for common health conditions

There is a group of 36 species used only for specific medicinal purposes (M) that are not used as food plants. These species are ingested only for specific health conditions. The lack of any usage as a health food is linked both to the taste and other sensory characteristics of products derived from these species but also to their often very strong pharmacological effects (for example as a purgative).

As reported in many studies (e.g. Heinrich et al., 1998; Cavero et al., 2013) dermatological conditions have the highest percentage of URs (3.3%, 21 species). The most popular species in this group are *Tilia platyphyllos* (0.8% of URs), *Chelidonium majus* (0.7%), *Verbena officinalis* (0.4%); *Rhamnus alaternus* (0.6%), *Plantago major* (0.3%) and *Cistus salviifolius* (0.3%). Interestingly, *Verbena officinalis* is also highlighted by Menendez-Baceta et al. (2014).

The second largest subgroup is “emotional problems” with 1.2% of the UR and four species employed; most notably *Tilia platyphyllos* (0.9%) and *T. cordata* (0.2%). Another key subcategories are respiratory conditions, (1.1%) with 9 species included, most importantly *Verbena officinalis* (0.5%). A range of plants is used for cleansing the body (0.80% of) and their use is limited to special health conditions, like for example purging with *Rhamnus alaternus* and *Centaurium erythraea* (0.3%). In the case of urogenital conditions (0.8%), *Equisetum arvense* (0.4%) and *Lepidium latifolium* (0.3%) stand out as the most important species. In the case of gastrointestinal conditions (28%), the most relevant species are *Parietaria judaica* (0.1%), used as a decoction to “cleanse the liver”, and *Illicium verum* and *Mentha pulegium* (0.07%, 4) that are used for its digestive properties. No species stands out in the small cardiovascular subgroup (0.30%), with *Rhamnus alaternus* (0.2%), *Verbena officinalis* and *Plantago major* (0.1%) each.

### 4.4 Food plants: ïdentifying key species for collected food consumption

We documented 73 locally collected species used only as a food and not having a medicinal report. The most popular species are *Castanea sativa* (2.2%), *Rubus castroviejoi* (1.7%), *Rubus caesius* (1.6%), *Fagus sylvatica* (1.6%), *Arbutus unedo* (1.3%) (Table 1).

Prepared foods include *Castanea sativa* (0.8%), *Cynara cardunculus* (0.2%), *Ruscus aculeatus* and *Sonchus oleraceus* (0.2% both). Salads are made with fresh leaves, adding vinegar, olive oil and salt. Some people bring all these elements to the field and when they find different species they prepare the salad in the field and eat it as a snack, or sometimes they eat them without preparations.

Only eight species were recorded as infusions without any medical claim (0.8%) most notably *Mentha aquatica* (0.2%), *Teucrium capitatum* and *Marrubium vulgare* (both 0.1%), highlighting that infusions generally are seen as a food with a medical purpose. Interestingly we only recorded very few reports for uses in jams, (0.3%), most importantly *Arbutus unedo* (0.1%, 4)*, Ribes rubrum, Ribes nigrum, Rubus castroviejoi* (0.1% each).

### 4.5 Plant parts and life forms

In the overall sample, fruits were used most commonly (33.7% of the URs), followed by dry inflorescences (13.6%), dry flowering aerial parts (7.2 %), flowering shoots (6.6%), with the remainder accounting for less than 6% of the total UR. Herbs are the most popular life form used accounting for 55.8% of all uses, followed by trees 28.1% and shrubs 16.2%. Fruits (18.5%) represent the most widely used plant part in the H-F group, followed by dried inflorescences, (13.5%) and dry flowering aerial parts (7.1%). In the group of local food plants (F), fruits (15.1%) were the part most frequently reported, followed by tender leaves (2.5%) and tender shoots (1.3%). In the case of medicinal plants (M), dry flowers (1.4%) represent the most widely used plant part; followed by fresh leaves and latex (both 1.0%).

### 4.6 Methods of preparation

Infusions (38.5%), crude plant materials (33.7% of the UR), decoctions (10.2%) and macerations (7.3%) are the most widely used methods. In case of preparations used both as a food and a medicine, infusions are the most popular form of preparation (33.9%), followed by raw plants (15.9%), boiling (6.9%), maceration (6.2%), roasted (2.1%), decoction and fried (1.8% both) and burning (0.4%).

Infusion is also the preferred method for M (4.0%), followed by direct application of the crude drug (2.0%), frying (1.1%), decoction (0.7%), boiling (0.4%), macerating (0.4%), and burning (0.1%). According to the 25 most important species, the species are prepared between eight and two different ways, pointing to a high degree of versatility of these species. Some species stand out for having a very versatile range of modes of preparation: *Sambucus nigra* and *Thymus vulgaris* (6 methods of preparations); *Urtica dioica, Quercus ilex* subsp. *ballota*, *Juglans regia*, and *Prunus spinosa* (5 each), in all cases linked to both their cultural importance as such and the diversity of uses they get.

The use of unprocessed fruits is of particular importance, with 15 species yielding fruits including berries and nuts such as *Sambucus nigra, Rubus ulmifolius, Castanea sativa,* and different species of acorns. All are consumed when they are ripe. A unique case is *Sorbus domestica* which needs to be ripened near the point when it becomes putrid (Table 1).

Another interesting group are chewable stems, leaves and barks called *masticantes* and used to obtain juices, fibres of the plants. For instance, the stems of *Rubus* species (called *carneros)* are peeled and chewed, the leaves of *Quercus ilex* subsp. *ballota* and *Fagus sylvatica*, are eaten or the seeds of *Triticum aestivum* are eaten immature and raw. The role of masticants as a source of phytochemicals has been previously highlighted (Johns et al., 1996, Leonti et al., 2006, Menendez-Baceta et al., 2012), since it might be related with the prophylactic effect of secondary chemicals.

The seeds of five species (0.7%) are roasted and ground into flour, sometimes mixed with corn flour (*Zea mays*) to be used for baking bread: *Quercus ilex* subsp. *ballota*, *Fagus sylvatica*, *Castanea sativa* and *Triticum aestivum*. Again this is a widespread tradition in Spain with a long history of use and species may be used interchangeably. In periods of scarcity, acorns were prepared as flour to make bread and other dishes (Triano et al., 1998, Blanco and Cuadrado, 2000, Fernández Ocaña, 2000, Tardío et al., 2006) a food use which can be traced back to prehistoric Spanish settlers (Tardío et al., 2006).

Other remarkable modes of preparation are jams and desserts (e.g. *Sambucus nigra, Rubus ulmifolius* and *Vaccinium myrtillus)*, and liqueurs (e.g. *Prunus spinosa, Sideritis hyssopifolia),* some of them marketed as quality local produce as in other Spanish regions (Pardo-de-Santayana et al., 2007).

The latex of several *Euphorbia* species is applied directly against verruca and moles but also as cheese rennet. Presently some families still collect the latex when it is very thick for preparing the cheese. They maintain the practice because “the taste is different” and it keeps the cheese for a longer time without it rotting. The need to find new tastes, new dishes, and to break the routine of the same dishes, having multiple preparations allows a wide variation in flavours for the same species.

### 4.7 The sources of locally used plants

An ongoing debate has focused on the zones where such resources are gathered. *Basoa* (forest and other uncultivated places further away from the farmhouse) account for 46.9% of the total UR, an interesting result since other studies (Frei et al., 2000, Stepp and Moerman, 2001, Kujawska and Pardo-de-Santayana, 2015) had shown that zones closes to the house tend to be the main sources of such products. Certain species that require social gatherings for their collection are usually found within this ecozone. When people collect plants they undertake other activities such as caring for animals, hunting and sharing time with other people. *Fincas* (cultivated fields) were the second most important zone (20.1%), followed by house garden (10.8%) and food garden (10.5%).

## 5 Conclusion

There is no sharp line dividing local food and medicine. This is a culturally constructed division and also influenced by environmental conditions, cultural background, traditional knowledge of the natural resources (useful plants in this case), education, economy, political movements, etc. (Collen et al 2015). From the analysis it also becomes apparent that these categories are dynamic. The preparations are characterized by having multiple methods of preparations and flexibility to use under subcategories of food and medicinal properties.

From the biogeographical location, it is clear that the Basque Country does not belong to the western Mediterranean region; however it shares with the latter a similar biodiversity and bio-cultural aspects. In comparison with other studies in the Mediterranean there are many similarities in the uses and preparations of different species of plants. Our study demonstrates that some of these plants are key Mediterranean species, used as food and medicine. Consequently, the study leads to new conceptual and practical implications in the way of understanding the meaning of Mediterranean regions, Mediterranean species, which involve more than the geographical location.

In all human cultures food diversity and diet are strongly linked to health. Such plant products may form a basis for developing novel useful products (like health foods) from this biocultural diversity. The present research provides baseline information that offers the possibility of further research into the traditional knowledge of the local people of the VIVA regions in the Basque Country. It is possible and necessary to maintain and further develop the information for future local and global uses which is a basis for conservation and sustaining and using these resources. If society’s desire is for new products which are sustainable, then much of what has been found in this research will provide the basis for potential new nutraceuticals. This information may even lead to economically profitable applications at a local, national and international level.

In the Basque Country further research on local and traditional knowledge regarding medicinal plants and food plants is needed. This study is just a stepping stone in trying to fulfil this need.

## Figures and Tables

**Table:** Medicinal and food uses of plants in VIVA area with three or more use-reports.

**Family Species**^a^	**Local names**	**Use-category and mode of use**
**Collection number**^b^		
**Comparison with other authors**^c^		

**APIACEAE**		
*Apium graveolens* L. †**;** 629	apio	*Clean the blood*: tender leaves and stems, boiled
T	*Vegetables*: tender leaves and stems, boiled alone or with other vegetables; boiled or raw, in salads
*Daucus carota* L.; 33	zanahoria	*Clean the blood*: roots, boiled
T	*Clean the skin*: seeds, macerated in olive oil, frictions
*Vegetables*: raw, alone or salads; boiled, stews
*Warts*: roots juice that rise when salt is added in a hole made at the top of the root, topical
*Foeniculum vulgare* Mill.; 620	anís, anís basto, hinojo	*Colds and sore throat*: leaves, infusion, alone or with *Thymus vulgari*s
T	*Digestive*: leaves, infusion, alone or with *Thymus vulgari*s; or with *Illicium verum*, *Mentha arvensis* and *Glycyrrhiza glabra*
*Vegetables*: young shoots, raw, snack, salads
*Heracleum sphondylium* L.; 673	hoja agria	*Vegetables*: tender leaves, raw, salads; soup
*Petroselinum crispum* (Mill.) Fuss; 133	perejil	*Conjunctivitis*: leaves, infusion, washings
*Seasoning*: leaves, flavour salads, meat, stews
**ARALIACEAE**		
*Hedera helix* L.; 774	hiedra	*Skin burns, cellulitis*: tender leaves and flowers, ointment, *pomada sanjuanera*⁎⁎
**ASTERACEAE**		
*Anacyclus clavatus* Pers.; 660	manzanilla, manzanilla de burro, manzanilla loca	*Herbal tea*: inflorescences, infusion
T	*Relaxant*: dry flowers, infusion
*Stomach pain:* inflorescences, infusion
*Arctium minus* (Hill) Bernh.; 579	bardana	*Vegetables*: tender leaves, boiled
T,PA
		
*Artemisia alba* Turra; 523	manzanilla	*Digestive, stomach pain*: flowering shoots, infusion
PA
		*Herbal tea*: flowering shoots, infusion
*Calendula officinalis* L.; 303	caléndula	*Skin inflammations or infections, burns, eczema*: inflorescences, oil infusion, topical; *infected wounds*: inflorescences, ointment, *pomada sanjuanera*⁎⁎
*Sweets*: inflorescences, raw
*Cichorium intybus* L. †; 212a	achicoria	*Coffee substitute*: roots, roasted and ground into flour, infusion
T,PA	*Vegetables*: leaves, raw, salads
*Cynara cardunculus* L. †; 30	cardo	*Vegetables*: leaf stalks and midribs, raw, boiled
*Cynara scolymus* L. †; 634	alcachofa, cardo	*Vegetables*: stem stalks and midribs, raw, boiled
*Helichrysum italicum* G. Don f.; 528	manzanilla, perpetua	*Digestive*: inflorescences, infusion
*Herbal tea*: inflorescences, infusion
*Liqueurs*: inflorescences, macerated in anisette with coffee and immature walnuts
*Helichrysum stoechas* (L.) Moench; 188	manzanilla, manzanilla perpetua	*Digestive*_:_ inflorescences, infusion alone or with *Chamaemelum nobile*
*Herbal tea*: inflorescences, infusion alone or with *Chamaemelum nobile*
Relaxant: inflorescences, infusion alone or with *Chamaemelum nobile*
*Respiratory problems, bronchitis, colds*: inflorescences, infusion alone or with *Chamaemelum nobile*
*Inula montana* L.; 148	árnica	*Skin infections, wounds*: flowering shoots, ointment, *pomada sanjuanera*⁎⁎
*Jasonia tuberosa* (L.) DC; 755	té de tierra	*Digestive*: flowering shoots, infusion
T	*Herbal tea*: flowering shoots, infusion
*Rheumatism and arthritis pain, bruises*: flowering shoots, ointment, *pomada sanjuanera*⁎⁎
*Lactuca sativa* L. †; 381	lechuga	*Vegetables*: leaves, raw in salad
*Matricaria discoidea* (Pursh) Rydb.; 614	manzanilla	*Digestive, stomach pain, sickness*: inflorescences, infusion
T
*Onopordum acanthium* L.; 166	cardo, toba	*Liver pain*: leaf stalks and midribs, boiled
T	*Vegetables*: tender leaves, soup
*Warts*: leaves, juice, topical
*Sonchus oleraceus* L.; 17	lechosino	*Vegetables*: tender leaves and stems, boiled with potatoes, soup; boiled, in salads
*Sonchus tenerrimus* L.; 260	lechosino	*Clean the stomach*: tender leaves, salad
*Kidney problems*: leaves, infusion
*Tanacetum corymbosum* (L.) Sch. Bip.; 245	manzanilla, tanaceto	*Herbal tea*: inflorescences, infusion
*Intestinal worms*: inflorescences, infusion
*Tanacetum vulgare* L. †; 355	manzanilla, matricaria cabeza grande	*Herbal tea*: inflorescences, infusion
T, PA	*Relaxant*: inflorescences, infusion
*Stomach pain*: inflorescences, infusion
*Taraxacum officinale* L.; 399	diente de león, lechosino	*Clean the blood*: leaves, infusion; *thin the blood*: leaves, boiled; *cholesterol lowering*: leaves, raw
M
*Clean the liver*: leaves, raw; infusion
*Vegetables*: leaves, raw, snack, salads
*Tussilago farfara* L.; 668	tusílago	*Skin inflammations and infections, wounds*: leaves, ointment, *pomada sanjuanera*⁎⁎
*Colds*: leaves, infusion
**BERBERIDACEAE**		
*Berberis vulgaris* L.; 122	agrace, agranzón	*Fruits*: raw
*Liqueur*: fruits, macerated in anisette
**BORAGINACEAE**		
*Borago officinalis* L.†; 64	borraja	*Clean the blood*: leaf stalks and midribs, boiled
T, PA.	*Coughs and colds*: flowers, infusion
*Vegetables*: leaf stalks and midribs, boiled, stews. Sometimes bottled
*Lithospermum officinale* L.; 592 PA.	té de bola, té de perla	*Herbal tea*: fructified aerial parts, infusion
		*Liver pain*: fructified aerial parts, infusion
*Pulmonaria longifolia* (L.) Huds.	pulmonaria	*Colds and bronchitis*: flowering aerial parts, infusion; fresh leaves, syrup
**BRASSICACEAE**		
*Brassica nigra* (L.) W.D.J. Koch†; 683	mostaza negra, nabo silvestre	*Vegetables*: leaves, raw, boiled in salad or with cabbage
*Brassica oleracea* L. †; 573	berza; *young shoot:* pámpano	*Vegetables*: young shoots, raw; leaves, boiled
*Lepidium latifolium* Medik.; 345	hoja del riñón, rompepiedra	*Kidney stones, cystitis*: leaves and flowers, infusion
*Rorippa nasturtium-aquaticum* (L.) Hayek; 540	berro	*Vegetables*: leaves, raw, salad
T, M.	*Clean the blood and body*: leaves, raw, salads
*Sinapis arvensis* L.; 125	nabo silvestre, sereu	*Vegetables*: leaves, raw, salad
**CAPRIFOLIACEAE**		
*Sambucus ebulus* L.; 216	yezgo	*Skin inflammations*: flowers, burnt, smoke bath
PA.
*Viburnum lantana* L.; 553	morrionera; *Fruit*: morrión	*Fruits*: raw
T
*Viburnum tinus* L.; 652	*Fruit*: marmaratila, masmarrade	*Fruits*: raw
**CARYOPHYLLACEAE**		
*Silene vulgaris* (Moench) Garcke; 287	colleja, molleja	*Vegetables*: tender leaves and stems, salted with egg; boiled
T
**CHENOPODIACEAE**		
*Beta vulgaris* var. *conditiva* Alef. †; 211	remolacha	*Clean and thin the blood*: roots boiled
*Vegetables*: roots, boiled, salad
*Beta vulgaris* var*. maritima* (L.) Moq. †; 219	acelga silvestre	*Diabetes*: young shoots, boiled, stews, soups
T	*Vegetables*: fresh shoots, dipper in batter, fried; boiled, stews
**CISTACEAE**		
*Cistus albidus* L.; 613	hierba lobera	*Skin infections, wounds*: aerial parts, decoction, washings
PA
*Cistus salviifolius* L.; 594	hierba lobera, jara, lobera	*Kidneys problems*: aerial parts, decoction
PA	*Internal ulcers*: aerial parts, decoction
*Skin infections*: aerial parts, decoction, washings
**CLUSIACEAE**		
*Hypericum perforatum* L.; 105	golondrina, flor de San Juan, hipérico, hierba de San Juan	*Clean the kidney*: flowering shoots, infusion, alone or with *Melissa officinalis* or *Cistus* sp.
T, PA
*Diarrhoea*: flowering shoots, infusion
*Insomnia, relaxant, sadness*: flowering shoots, infusion with *Melissa officinalis; depression*: inflorescence, oil infusion, frictions
*Herbal tea*: flowering shoots, infusion, alone or with *Melissa officinalis*
*Skin infections and inflammations*: flowering shoots, ointment, *pomada sanjuanera*⁎⁎
**CONVOLVULACEAE**		
*Convolvulus arvensis* L.; 671	amapola de carihuela	*Vegetables*: tender leaves and flowers, raw
T
**CRASSULACEAE**		
*Hylotelephium telephium* (L.) H.Ohba†; 405	bálsamo, siempreviva	*Infected wounds, skin inflammation*: peeled leaves (without epidermis), topical
*Sedum spurium* M. Bieb.; 729	bálsamo	*Infected wounds, skin inflammations*: peeled leaves (without epidermis), topical
*Umbilicus rupestris* (Salisb.) Dandy; 342	bálsamo, campanilla, chupamateria, hierba callera, ombligo de Venus, sacamateria	*Infected wounds, skin inflammations, corns, hard skin, warts*: peeled leaves (without epidermis), topical
*Vegetables*: leaves, raw, salads; boiled with *Urtica dioica*, *Medicago sativa* and other vegetables, soups, sometimes bottled
**CUCURBITACEAE**		
*Cucurbita ficifolia* Bouche.†; 365	calabaza, calabazo, calabaza de cabello de ángel	*Fruits*: jam (*cabello de ángel*), boiled with sugar
*Vegetables*: tender stems, seeds raw; flowers, dipper in batter, fried
**CUPRESSACEAE**		
*Juniperus communis* L.; 49	enebro	*Digestive*: cones, raw
T, PA	*Earache*: cones, almond oil infusion, warmed in a bain marie, ear drops
*Liqueur*: cones, macerated in anisette, liqueur or gin
*Rheumatism and arthritis pain*: cones, oil infusion with *Rosmarinus officinalis,* frictions
*Skin infections*: cones, ground, poultice; *warts*: cones, boiled with salt, compresses; same number of cones as warts in a piece of cloth, buried in the ground
*Juniperus oxycedrus* L.; 546	abrojo, enebro, ginebro	*Liqueur*: cones, macerated in anisette, liquor or gin
T
*Juniperus phoenicea* L.; 249	abrojo romero, sabina	*Warts*: cones and branches, boiled with salt, compresses
**DIOSCOREACEAE**		
*Tamus communis* L.; 656	uva de perro	*Rheumatism and arthritis pain*: fruits, raw, frictions
T, M, PA
**EQUISETACEAE**		
*Equisetum arvense* L.; 733	cola de raposo, cola de ratón, cola de zorro	*Bruises*: aerial parts, ground, poultice
PA.	*Clean the blood*: fertile aerial parts, decoction with *Taraxacum officinale* leaves
*Diuretic, kidney problems*: aerial parts, infusion
**ERICACEAE**		
*Arctostaphylos uva-ursi* (L.) Spreng.; 200	gayuba	*Prostate inflammations, cystitis*: fruits and leaves, infusion; *kidney infections, diuretic*: fruits and leaves, infusion with *Erica vagans*
T
*Fruits*: raw
*Erica vagans* L.; 460	brezo	*Herbal tea*: flowering aerial parts, infusion
*Vaccinium myrtillus* L.	arándano, ráspano, rámpano	*Fruits*: raw
T, M, PA
**EUPHORBIACEAE**		
*Euphorbia peplus* L.	lechetrezna	*Warts*: latex, topical
*Euphorbia serrata* L.	lechetrezna	*Warts*: latex, topical
T
*Euphorbia villosa* Waldst. & Kit. ex Willd.; 771	lechetrezna	*Warts*: latex, topical
*Euphorbia* sp.*; 565*	lechetrezna	*Cheese rennet*: latex
*Warts*: latex, topical
**FABACEAE**		
*Glycyrrhiza glabra* L. †; 789	regaliz	*Herbal tea*: aerial parts and roots, infusion
T	*Digestive*: aerial parts and roots, infusion; stem chewed
*Lathyrus cicera* L. †; 651	arverjilla, guisante silvestre	*Herbal tea*: aerial parts, infusion, alone or with *Mentha arvensis*
*Medicago sativa* L.; 222	alfalfa	*Clean the blood*: young shoots, with water
T	*Depression*: young shoots, infusion, alone or with milk
*Herbal tea*: young shoots, infusion
*Vegetables*: young shoots, raw; soup with *Urtica dioica*, *Umbilicus rupestris* and other vegetables
*Phaseolus vulgaris* L. †	alubia	*Diabetes*: seeds, infusion
*Fruits*: seeds, stews
*Trigonella foenum-graecum* L. †	alholva	*Vegetables:* fruits, shoots, raw, soups, stews
*Pisum sativum* L. †; 328	guisante	*Vegetables*: seeds, soups, stews
*Vicia faba* L. †; 220	haba	*Anaemia*: immature seeds, boiled; seeds, toasted and ground, infusion
*Coffee substitute*: seeds, toasted and ground into flour, infusion
*Vegetables*: immature seeds, boiled and salted or puree
**FAGACEAE**		
*Quercus faginea* Lam.; 44	roble encina; *leave*: pasto de roble	*Coffee substitute*: fruits, toasted and ground into flour, infusion
T, M	*Flavouring cheese*: fruits, olive oil infusion with *Juniperus communis* cones and *Lavandula latifolia*
*Fruits*: raw, toasted
*Skin infections, wounds*: bark, poultice
*Quercus faginea x Q. pyrenaica;* 516	roble	*Coffee substitute*: fruits, toasted and ground into flour, infusion
*Fruits*: raw, toasted
*Herbal tea*: leaves and fruits, infusion
*Vegetables*: leaves, chewed
*Quercus ilex* L. subsp. *ilex*; 515	encina	*Coffee substitute*: fruits, toasted and ground into flour, infusion
T, M	*Fruits*: raw, toasted
*Quercus* sp.*;* 676; 158	roble	*Coffee substitute*: fruits, toasted and ground into flour, infusion
*Vegetables*: leaves, chewed
**GENTIANACEAE**		
*Centaurium erythraea* Rafn; 479	centaura, centaurea menor, sanguinaria	*Clean the blood*: flowering shoots, infusion
T	*Depression*:flowering shoots, infusion
*Digestive*: flowering shoots, infusion
*Gentiana acaulis* L.	pan de cuco	*Vegetables*: flowering shoots, raw
**GROSSULARIACEAE**		
*Ribes alpinum* L.; 543	grosellero; *fruit*: abubilla, agrace, grosella	*Fruits*: raw
T, PA
*Ribes nigrum* L†.; 683	grosellero; *fruit*: grosella	*Fruits*: raw; jam
PA
*Ribes rubrum* L. ; 370	aranzón, *fruit*: grosella, grosella roja, uvín, uvín rojo	*Fruits*: raw; jam
T
*Ribes uva-crispa* L.; 727	uva espín	*Fruits*: raw
T
**ILLICIACEAE**		
*Illicium verum* Hook. f.†; 773	anís	*Digestive*: fruis, infusion, alone or with *Foeniculum vulgare* and *Glycyrrhiza glabra*; liqueur, fruits macerated in anisette with *Prunus spinosa* fruits
**LAMIACEAE**		
*Calamintha nepeta* (L.) Savi; 440	menta, menta del monte, orégano del monte	*Herbal tea*: aerial parts, infusion
T	*Relaxant*: aerial parts, infusion
*Lavandula latifolia* Medik.; 276	lavanda	*Relaxant*: inflorescences, infusion; flowering shoots, oil infusion, frictions
PA	*Flavouring cheese*: inflorescences, olive oil infusion with *Juniperus communis* cones and acorns
*Skin infections*: flowering shoots, ointment, *pomada sanjuanera*⁎⁎
*Marrubium vulgare* L.; 596	menta hedionda	*Herbal tea*: aerial parts, infusion
PA
*Melissa officinalis* L. ††; 474	melisa	*Bronchitis*: aerial parts, infusion
T, PA	*Herbal tea*: aerial parts, infusion, alone or with *Hypericum perforatum*
*Relaxant, depression*: aerial parts, infusion, alone or with *Hypericum perforatum*
*Stomach pain*: aerial parts, infusion
*Mentha aquatica* L.; 719	menta, menta poleo	*Herbal tea*: leaves, infusion
T, M, PA	*Seasoning:* leaves, flavour rice pudding, meat, soups
*Mentha arvensis* L.; 721	hierbabuena, hierba santa, menta	*Herbal tea*: leaves, infusion, alone or *Lathyrus cicera*
PA	*Seasoning:* leaves, flavour rice pudding
*Mentha x gentilis* L. †; 296	hierbabuena, menta	*Herbal tea*: leaves, infusion
*Relaxant*: leaves, infusion
*Mentha longifolia* (L.) Huds.	hierba santa, menta silvestre	*Digestive*: leaves, infusion
T	*Seasoning:* leaves, flavour rice pudding
*Mentha x piperita* L. †; 123	hierbabuena, hierba santa, menta	*Digestive, stomach pain*: leaves, infusion
*Herbal tea*: leaves, infusion
*Relaxant*: leaves, infusion
*Seasoning:* leaves, flavour rice pudding
*Mentha pulegium* L.; 265	batán, menta lombricera, menta poleo	*Digestive, stomach pain*: leaves, infusion; liqueur, macerated in anisette with *Prunus spinosa* fruits, *Rosmarinus officinalis* and *Thymus vulgaris*
T, PA
*Intestinal worms*: leaves, infusion
*Mentha spicata* L.; 338	hierbabuena	*Digestive, stomach pain*: leaves, infusion
T	*Herbal tea*: leaves, infusion
*Relaxant*: leaves, infusion
*Seasoning*: leaves, flavour meat, soups, rice pudding
*Ocimum basilicum* L.††; 407	albahaca	*Digestive*: leaves, infusion with *Thymus vulgaris*
*Relaxant*: leaves, infusion, alone or with *Thymus vulgaris*
*Seasoning*: leaves, flavour, meat, pasta, stews
*Phlomis lychnitis* L.; 598	candilera	*Clean the stomach*: flowering shoots, infusion
T	*Herbal tea*: flowering shoots, infusion
*Salvia lavandulifolia* Vahl; 332	salvia	*Herbal tea*: leaves, infusion
T, PA	*Relaxant*: leaves, infusion
*Seasoning*: leaves, flavour meat, stews
*Teucrium capitatum* L.; 599	sanguinaria	*Herbal tea*: flowering shoots, infusion
T, PA
*Thymus mastichina* (L.) L.; 9	tomillo	*Colds and respiratory problems*: flowering shoots, infusion with lemon
T
*Thymus praecox* Opiz; 600	orégano silvestre	*Herbal tea*: flowering shoots, infusion
T	*Headache*: flowering shoots, infusion
*Thymus zygis* L.; 172	tomillo	*Colds and chest infections*: flowering shoots, infusion
T	*Seasoning*: leaves, flavour meat, stews
**LAURACEAE**		
*Laurus nobilis* L. †; 128	laurel	*Digestive*: leaves, infusion
M	*Seasoning*: leaves, flavour meat, sausages, snails, fish, stews
**LILIACEAE s.l. (incl. Alliaceae, etc.)**		
*Allium ampeloprasum* L.; 562	ajete, ajo silvestre, puerro, puerro silvestre	*Seasoning*: bulb and pseudostem, flavour meat, stews soups
*Allium cepa* L. †; 335	cebolla	*Seasoning*: flowers, bulb and pseudostem, flavour meat, stews, soups
*Skin inflammations and infections*: second layer of the bulb, heated, poultice; *skin burns*: first layer of the bulb, fried, put in a piece of cloth, poultice
*Allium porrum* L. †; 235	puerro	*Vegetables*: pseudostems, boiled, stews
*Allium sativum* L. †; 334	ajo	*Colds and phlegm*: bulbs, raw
*Digestive*: bulbs, macerated in olive oil
*Flavouring*: bulbs, flavour, meat, stews
*Prevent infections*: bulbs, soup with bread (*sopa de ajo*)
*Rheumatism and arthritis pain*: bulbs, macerated in olive oil
*Vegetables*: bulbs, raw
*Asparagus acutifolius* L.; 236	esparraguera; *young shoot*: espárrago, espárrago silvestre	*Vegetables*: young shoots, raw
T, PA
*Asparagus officinalis* L.	*young shoot*: espárrago	*Vegetables*: young shoots, raw
PA
*Merendera montana* (L.) Lange; 405	quitameriendas	*Seasoning*: stamen, flavour rice (colourant)
T
*Ruscus aculeatus* L.; 571T	rusco	*Vegetable*: young shoots, raw; boiled or salted
**MALVACEAE**		
*Althaea officinalis* L.; 454	malvavisco	*Colds*: flowering shoots, infusion; roots, syrup
T, PA
*Malva moschata* L.; 437	malva; *fruit*: quesito	*Colds*: flowering shoots, infusion
*Fruits*: immature fruit, raw
*Malva neglecta* Wallr.; 438	malva; *fruit*: quesito	*Colds*: flowering shoots, infusion
T, PA	*Fruits*: immature fruit, raw
**MYRTACEAE**		
*Eucalyptus globulus* Labill**.;** † 780	eucalipto	Colds and sore throat: leaves, infusion, alone or with lemon; boiled, inhalations
**MORACEAE**		
*Ficus carica* L. †; 778	*Fruit*: higo	*Fruits*: raw
M
**OLEACEAE**		
*Ligustrum vulgare* L.	aligustre	*Fruits*: raw
**PAPAVERACEAE**		
*Chelidonium majus* L. ; 115	berruguina, celidonia, flor del yodo, golondrina	*Bruises*: flowering aerial parts boiled
PA	*Relaxant*: flowering aerial parts
*Warts*: latex, topical
**PINACEAE**		
*Pinus sylvestris* L.; 560	pino	*Colds*: shoots, infusión
*Fruits*: seeds, raw, toasted
**PLANTAGINACEAE**		
*Plantago lanceolata* L.; 288	llantén, llantén macho, llantén menor	*Skin inflammations*: leaves, raw, poultice; *corns*: leaves, ointment, *pomada sanjuanera*⁎⁎
T, M, PA
*Swollen areas*: leaves, decoction, washings
*Plantago major* L.; 384	llantén, llantén hembra, llantén femenino	*Skin infections*: leaves, infusion, washings; *infected wounds*: leaves, crushed in a cloth, poultice; *bites*: leaves, poultice; *herpes*: ointment, *pomada sanjuanera*⁎⁎
T, PA
*Kidney problems*: leaves, infusion
*Piles*: leaves, crushed in a cloth, poultice; ointment, *pomada sanjuanera*⁎⁎
*Digestive*: leaves, infusión
**POACEAE**		
*Hordeum vulgare* L. †; 776	cebada	*Coffee substitute*: fruits toasted and ground into flour, infusion
*Triticum aestivum* L. †	trigo	*Fruits*: immature fruits, raw
*Flour*: fruits, ground into flour, bread
*Zea mays* L. †	maíz	*Flour*: fruits, ground into flour, bread (*talo or moroqui*)
**POLYGONACEAE**		
*Rumex* sp.	acedera, achitabla	*Vegetables*: tender leaves, raw, salad
**PRIMULACEAE**		
*Anagallis arvensis* L.; 627	muraje	*Skin inflammations and infections, burns, corns, wounds*: leaves and flowers, ointment, *pomada sanjuanera*⁎⁎
PA
*Primula veris* L.; 283	flor de mayo, flor de San José, pámpano	*Sweets*: flowers, nectar, sucked
*Skin inflammations*: leaves and flowers, ointment, *pomada sanjuanera*⁎⁎
*Vegetables*: tender leaves, raw
**RANUNCULACEAE**		
*Ranunculus ficaria* L.; 527	celidonia menor	*Burns*: flowers and leaves, ointment, *pomada sanjuanera*⁎⁎
**RHAMNACEAE**		
*Rhamnus alaternus* L.; 375	carrasquilla	*Colds, sore throat*: flowering branches, infusion
T, PA	*High blood pressure, thin the blood*: flowering branches, infusion
**ROSACEAE**		
*Amelanchier ovalis* Medik.; 552	guillomo, alcurnio; *fruit*: arándano silvestre, manzana silvestre	*Fruits*: raw, jam
T
*Fragaria x ananassa* (L.) Mill. †; 125	*Plant and fruit*: fresa, marrubio	*Fruits*: raw
*Crataegus monogyna* Jacq. ; 698	espino albar, espino blanco, *fruit*: anrolica, enrolica, manzana del pastor	*Constipation*: flowering branches, infusion
T,M	*Fruits*: raw
*High blood pressure, thins the blood*: flowering branches, infusion
*Relaxant*: flowering branches, infusion, alone or with *Melissa officinalis*
*Malus domestica* L. †; 312	manzano; *fruit*: manzana	*High blood pressure, thins the blood*: flowering branches, infusion
*Relaxant*: flowering branches, infusion, alone or with *Melissa officinalis*
*Malus sylvestris* Mill.; 738	manzano silvestre; *fruit*: gustincha, maguilla, maguillo, maila, majilla, manzana silvestre	*Constipation*: flowering branches, infusion
T, M	*Fruits*: raw
*Liqueur*: fruits, macerated in anisette or liquor, alone or *Chamaemelum nobile*
*Mespilus germanica* L.; 785	níspero	*Fruits*: raw
T, M
*Potentilla reptans* L.; 101	cinco en rama	*Piles:* leaves in a piece of cloth until dry
PA	*Skin infections, wounds*: flowering shoots, ointment, *pomada sanjuanera*⁎⁎
*Prunus avium* (L.) L.; 227	cerezo silvestre; *fruit*: cereza	*Fruits*: raw
T, M	*Liqueur*: fruits, macerated in anisette
*Prunus insititia* L.; 314	cerezo silvestre, ciruelo silvestre; *fruit*: arañone	*Fruits*: raw
T, M
*Prunus mahaleb* L.; 539	cerezo de mahoma, ciruelo silvestre	*Fruits*: raw
T
*Pyrus communis* L.†; 228	peral; *fruit*: pera	*Fruits*: raw, fruit salad, salad
*Rosa canina* L.; 697	escalambrujo, rosal, tapaculo; *young shoot*: carnero, mato	*Fruits*: raw
T	*Liqueur*: fruits, macerated in anisette
*Vegetables*: young shoots, peeled, raw
*Rosa* sp.; 281	calambrujo, rosa silvestre, tapaculo; *fruit*: arañón, carajachi, escalambrujo; *young shoot*: carnero, mato	*Colds and bronchitis*: fruits, decoction
*Fruits*: raw, boiled with sugar
*Vegetables*: young shoots, peeled, raw
*Rubus idaeus* L.; 730	frambuesa; *young shoot*: carnero, mato	*Fruits*: raw
T	*Vegetables*: young shoots, peeled, raw
*Rubus* sp.*;* 301	zarza; *fruit*: mora; *young shoot*: carnero, mato	*Fruits*: raw
*Vegetables*: young shoots, peeled, raw
*Sanguisorba minor* Scop.; 424	pimpinela	*Vegetables*: tender leaves, raw, snack, salad
T
*Sorbus aria* Crantz; 511T	mostajo; *fruit*: manzanica silvestre	*Fruits*: raw
**RUBIACEAE**		
*Galium aparine* L.; 630	amor de hortelano, lampazo	*Vegetables*: tender aerial parts, raw
**RUTACEAE**		
*Citrus limon* (L.) Osbeck †	limonero; *fruit*: limón	*Colds, sore throat*: leaves, infusion with lemon juice or *Eucalyptus globulus*
		*Stomach pain, diarrhoea*: fruits, juice
**SCROPHULARIACEAE**		
*Veronica* sect. *beccabunga* Griseb. ; 577	berra, berraña	*Vegetables*: tender leaves, raw; boiled, salads
M
*Scrophularia alpestris* Gay ex Benth.; 717	hierba mora, lengua de perro	*Skin infections, burns*: leaves, raw with olive oil or heated with oil, poultice
*Verbascum thapsus* (L.); 156	gordolobo	*Chest infections*: flowers, infusion, syrup
Colds, cough, asthma: flowers, infusion
*Earache*: flowers, olive oil infusion, ear drops
**TAXACEAE**		
*Taxus baccata* L.; 325	tejo	*Fruits*: aril, raw
T, M, PA
**TILIACEAE**		
*Tilia cordata* Mill.; 452	tila	*Relaxant*: flowers, infusion
T
*Tilia platyphyllos* Scop.; 558	tila	*Relaxant*: flowers, infusion
T	*Toothache*: flowers, infusion
**URTICACEAE**		
*Parietaria judaica* L.; 561	parietaria	*Liver pain and clean*: aerial parts, infusion
*Urtica dioica* L.; 153	ortiga	*Circulatory conditions, thin the blood:* tender leaves and stems, boiled, soup; raw, frictions
T, M
*Gall stones*: tender leaves and stems, boiled, soup
*Herbal tea*: aerial parts, infusion
*Rheumatism and arthritis pain*: tender leaves and stems, boiled, soup; raw, frictions
*Stimulate immune system*: tender leaves and stems, boiled, soup
*Vegetables*: tender leaves and stems; boiled, soup, stews, salted
*Urtica urens L. ;* 117	ortiga	*Circulatory problems, clean the blood:* tender leaves and stems, boiled, soup; raw, frictions
T
*Vegetables*: tender leaves and stems; boiled, soup
**VERBENACEAE**		
*Verbena officinalis* L.	verbena	*Colds, sore throat*: aerial parts, decoction; *sinusitis*: aerial parts, fried with egg, poultice
PA
*‘Cholesterol-lowering*: aerial parts, decoction
*Skin infections and inflammations*: aerial parts, decoction, washings; aerial parts, ointment, *pomada sanjuanera*⁎⁎
**VITACEAE**		
*Vitis vinifera* L. †; 787	vid; *fruit*: uva	*Vegetables*: Young shoots, raw
T

For symbols and acronyms, see also Table 1.

^⁎^*Patxaran: Prunus spinosa* fruits are macerated in anisette for five or six months with coffee beans, cinnamon bark and *Chamaemelum nobile* inflorescences.

⁎⁎ Pomada sanjuanera: Ointment prepared frying together many plants such as: *Achillea millefolium*, *Allium ampeloprasum*, *Anagallis arvensis*, *Calendula officinalis*, *Chamaemelum nobile*, *Cruciata glabra*, *Eleocharis quinqueflora*, *Glycyrrhiza glabra*, *Hedera helix*, *Hypericum hirsutum*, *Hypericum perforatum*, *Inula montana*, *Jasonia tuberosa*, *Lavandula latifolia*, *Malva sylvestris*, Mentha×piperita, *Pinus sylvestris*, *Plantago lanceolata*, *Plantago major*, *Potentilla erecta*, *Potentilla reptans*, Primula veris, *Pulmonaria longifolia*, *Ranunculus ficaria*, Rumex sp., *Sambucus nigra*, *Santolina chamaecyparissus*, *Symphytum tuberosum*, *Thymus vulgaris*, *Tussilago farfara*, *Verbascum thapsus* and *Verbena officinalis*.^⁎⁎⁎^ Olives in brine:olives are macerated in water with salt until the bitter flavour has left.Then they are macerated in cold water, garlic,thyme and rosemary for two months.

a Unless indicated by a cross (†) all species are non-cultivated; species which are both cultivated and non cultivated are indicated with two crosses (††).

b Collector: Rocío Alarcón.

c Comparison with other authors: T (Tardío et al., 2006), M (Menendez-Baceta et al., 2012), PA (Puente Amestoy, c. 1960).

**Table 1:** Top 25 food and medicinal species according to the number of use-reports.

**Species**^a^	**Local names**	**Use-category and mode of use**	**Number of use-reports**	**Use-category number of use-reports (percentage of total)**^d^
**Family; collection number**^b^				
**Comparison with other authors**^c^				

*Jasonia glutinosa* (L.) DC.	té, té de monte, té de roca, té de puerto	*Herbal tea:* flowering shoots, infusion, alone or with *Chamaemelum nobile, Illicium verum, Prunus spinosa, Thymus vulgaris* or with milk	269	H-F 375 (6.63%)
Asteraceae; 593
T
*Digestive, stomach pain, diarrhoea:* flowering shoots, infusion	100
*Liqueur:* flowering shoots, macerated in anisette	6
*Chamaemelum nobile* (L.) All.	manzanilla, manzanilla amarga, manzanilla de altura, manzanilla de la sierra, manzanilla del monte, manzanilla fina, manzanilla pequeñita, manzanilla real	*Digestive, stomach pain, sickness*: inflorescences, infusion, liqueur, *patxaran*⁎	165	H-F 352 (6.22%)
Asteraceae; 743
T, PA	*Herbal tea*: inflorescences, infusion, alone or with anisette, *Malva sylvestris*, *Thymus vulgaris* or *Helichrysum stoechas*	147
*Relaxant*: inflorescences, infusion, alone or with *Malva sylvestris* or *Helichrysum stoechas*	22
*Headache*: inflorescences, infusion	7
*Skin inflammation, infection*: inflorescences, ointment, *pomada sanjuanera*⁎⁎	5
*Clean the body*: inflorescences, infusion	3
*Liqueur*: inflorescences, *patxaran*⁎	3
*Prunus spinosa* L.	*fruit*: arán, escaramujo, endrino, arañón, churli, ciruela, endrina, carcarachi	*Liqueur*: fruits, *patxaran*⁎	88	H-F 268 (4.74%)
Rosaceae; 322	*Fruits:* raw, jam or syrup with cinnamon	75
T, M, PA	*Colds and coughs, sore throat*: fruits, decoction, drunk and gargles; bark, gargles	45
*Digestive, stomach pain, diarrhoea*: fruits, decoction; fruits, liqueur, *patxaran*⁎	38
*Infected wounds*; bark, washings	3
*Premenstrual pain*; fruits, liqueur, *patxaran*⁎	3
*Quercus ilex* subsp. *ballota* (Desf.) Samp.	carrasca, encina; *fruit*: bellota	*Fruits:* raw, toasted	105	H-F 242 (4.28%)
*Coffee substitute:* fruits, toasted and ground into flour, infusion	88
Fagaceae; 472
T
*Liqueur*: fruits, macerated in anisette	20
*Flour:* fruits, ground into flour, bread and cakes	9
*Herpes:* flowers, infusion, sometimes with *Juglans regia* leaves, washings; *wounds*: bark, decoction, washings	5
*Clean the body*: roots and bark, infusion	4
*Vegetables:* leaves, chewed	3
*Santolina chamaecyparissus* L.	manzanilla, manzanilla de buey, manzanilla de burro, manzanilla de caballo, manzanilla basta, manzanilla de campo, manzanilla gorda, santolina	*Digestive, stomach pain*: inflorescences, infusion	108	H-F 210 (3.71%)
Asteraceae; 352
*Herbal tea*: inflorescences, infusion	85
T,PA
*Ingrown nails*: aerial parts, boiled and left macerating in water, washings; boiled and smashed, topical; *skin infections*: inflorescences, ointment, *pomada sanjuanera*⁎⁎	10
*Relaxant*: inflorescences, infusion	4
*Hair loss*: inflorescences, infusion, washings	3
*Thymus vulgaris* L.	tomillo	*Colds and chest infections, sore throat*: flowering shoots, infusion, alone or with honey, lemon or *Foeniculum vulgare*	42	H-F 168 (2.97%)
Lamiaceae; 111
*Seasoning*: flowering shoots, flavour meat, olives⁎⁎⁎	36
T
*Herbal tea*: flowering shoots, infusion	33
*Skin inflammation and infections:* flowering shoots, infusion, washings; ointment, *pomada sanjuanera*⁎⁎; *mouth infections:* flowering shoots, infusion, washings	13
*High blood pressure, clean the blood, heart problems, fluid retention*: flowering shoots, infusion, alone or with honey**,** lemon, *Rosmarinus officinalis* or with *Jasonia glutinosa* and *Chamaemellum nobile*	25
*Digestive*: flowering shoots, infusion with *Ocimum basilicum*	5
*Rheumatism and arthritis*: flowering shoots, tincture in cologne	7
*Cystitis*: flowering shoots, infusion with *Chamaemelum nobile*	4
*Relaxant*: flowering shoots, infusion	3
*Sambucus nigra* L.	sabuco, saúco	*Colds, bronchitis, sore throat*: flowers, infusion, alone or with *Malva sylvestris;* flowering branches, burnt, smoke inhalations; stems, fruits; steam inhalations; *asthma and chest infections*: flowers, fried with egg, eaten	87	H-F 156 (2.76%)
Caprifoliaceae; 86
T
*Eczema, skin inflammation and infections, wounds*: leaves, cortical parenchyma (“inner bark”), ointment, *pomada sanjuanera*⁎⁎; flowering branches, ointment with *Rosmarinus officinalis*	18
*Fruits*: raw, jam	18
*Clean the body*: flowers, infusion	4
*Herbal tea*: flowers, infusion	8
*Stomach pain*: flowers, infusion	7
*Liqueur*: fruits, macerated in anisette	4
*Headache*: flowers, fried with egg, eaten	3
*Toothache (cavities)*: flowering branches, burnt, smoke baths	3
*Vegetables:* flowers, omelette	4
*Juglans regia* L. †	nogal; *fruit*: nuez, *inmature fruit:* cucón	*Fruits*: raw, toasted	83	H-F 156 (2.76%)
Juglandaceae; 383	*Clean the body*: leaves, infusion	24
M	*Liqueur*: immature fruits, macerated in anisette alone or with *Chamaemelum nobile*	14
*External infections, wounds, mouth ulcers, verrucae and moles, herpes:* leaves, infusion, alone or with *Quercus ilex* subsp. *ballota* flowers, washings	12
*Prevent high cholesterol, heart problems:* fruits, raw; immature fruits and young shoots, liqueur	7
*Rheumatism and arthritis*: immature fruits alone or with young shoots, liqueur in anisette, alone or with *Chamaemelum nobile*, drunk, frictions	6
*Intestinal worms*: leaves, infusion	4
*Vaginal infections*: leaves, infusion, vaginal washings	3
*Digestive*: fruits, raw; liqueur, immature fruits, liqueur in anisette with *Chamaemelum nobile*	3
*Rubus ulmifolius* Schott	mora, zarzamora; *fruit*: mora del alto; *young shoot*: carnero, chispío, mato	*Fruits*: raw, jam	100	H-F 144 (2.55%)
Rosaceae; 118	*Vegetables*: young shoots, peeled, raw	31
T, M
*Sore throat, colds and respiratory problems:* flowers and tender shoots, infusion	10
*Liqueur:* fruits, macerated in anisette	3
*Anthemis arvensis* L.	manzanilla, manzanilla basta, manzanilla bastarda, manzanilla de vacas, manzanilla de burro, manzanilla dulce, manzanilla falsa	*Stomach pain, digestive* Inflorescences, infusion	62	H-F 113 (2.00%)
Asteraceae; 362
*Herbal tea*: inflorescences, infusion	32
*Relaxant*: inflorescences, infusion	8
*Conjunctivitis*: inflorescences, decoction, washings	7
*Piles*: inflorescences, infusion, washings	4
*Castanea sativa* Mill. †	castaño; *fruit*: castaña	*Fruits*: raw, boiled, alone or in stews	101	F 125 (2.21%)
Fagaceae; 134	*Coffee substitute*: fruits, toasted and ground into flour, infusion	21
T,M
*Flour*: fruits, ground into flour, bread	3
*Sorbus domestica* L. ††	gerbal, jurbal, *fruit*: gerbal, poma	*Fruits:* raw; jam, alone or with apples	104	H-F107 (1.89%)
Rosaceae; 607	*Diarrhoea:* fruits, raw	3
T
*Malva sylvestris* L.	malva	*Coughs and colds, sore throat*: flowers, infusion, alone or with *Origanum vulgare* or *Sambucus nigra;* leaves, decoction; *bronchitis and asthma*: leaves, decoction, steam inhalations	57	H-F 95 (1.68%)
Malvaceae; 292
T,M,PA
*Fruits*: raw	19
*Herbal tea*: flowers, infusion, alone or with *Chamaemellum nobilis*	6
*Skin inflammations*: flowers and leaves, ointment, *pomada sanjuanera*⁎⁎	4
*Relaxant*: flowers, infusion with *Chamaemellum nobilis*	3
*Swollen legs (fluid retention)*: flowers and leaves, decoction, washings	3
*Eye inflammation*: flowers and leaves, decoction, eye washings	3
*Rubus castroviejoi*	mora, zarzamora, zarza; *young shoot*: mato	*Fruits*: raw, jam	58	F 97 (1.71%)
Monasterio-Huelin	*Vegetables*: young shoots, peeled, raw	39
Rosaceae; 168
*Rubus caesius* L.	mora rastrera, zarza, zarzamora; *fruit*: mora; *young shoot*: carnero, chispío	*Fruits*: raw	54	F 89 (1.57%)
Rosaceae; 426	*Vegetables*: young shoots, peeled, raw	35
T
*Fagus sylvatica* L.	obe, haya; *fruit*: hayuco	*Fruits*: raw	69	F 88 (1.56%)
Fagaceae; 195	*Vegetables*: leaves, raw, chewed	11
T,M	*Flour*: fruits, toasted and ground into flour, mixed with wheat or other cereals, bread	8
*Tanacetum parthenium* (L.) Sch. Bip.†	moriza, manzanilla, manzanilla de huerta, manzanilla de jardín	*Digestive, stomach pain*: flowering shoots, infusion	37	H-F 77 (1.36%)
Asteraceae; 354	*Herbal tea*: flowering shoots, infusion	24
*Relaxant*: flowering shoots, infusion	16
*Arbutus unedo* L.	abis, bordo, borto, burrubiote, madroño	*Fruits*: raw, jam	71	F 74 (1.31%)
Ericaceae; 90	*Liqueur*: fruits, macerated in anisette	3
T,M,PA
*Rosmarinus officinalis* L.	romero	*Seasoning*: flowering shoots, flavour meat, olives⁎⁎⁎	25	H-F 73 (1.29%)
Lamiaceae; 26
*Rheumatism and arthritis, body pains*: flowering shoots, tincture in alcohol or oil infusion with *Cupressus* sp., frictions	16
T,PA
*Sore throat, colds and phlegm*: flowering shoots, infusion, alone or with honey	12
*Relaxant*: flowering shoots, infusion	10
*Herbal tea*: flowering shoots, infusion	10
*Achillea millefolium* L.	cien flores, manzanilla, milflores, milenflora, milenrama	*Digestive, stomach pain, diarrhoea*: flowering shoots, infusion, alone or with *Lythrum salicaria*	27	H-F 71 (1.25%)
Asteraceae; 18	*Herbal tea*: flowering shoots, infusion	14
*Piles*: flowering shoots, put in a piece of cloth and kept in the trousers’ back pocket until dry	11
*Rheumatism and arthritis pain*: flowering shoots, ointment, leaves and flowers *pomada sanjuanera*⁎⁎	8
*Circulatory problems, clean the blood*: flowering shoots, infusion	7
*Headache:* flowering shoots, bag left under the pillow, inhalations	4
*Corylus avellana* L.	avellano; *fruit*: avellana	*Fruits*: raw, toasted	59	F 68 (1.20%)
Betulaceae; 379	*Liqueur:* fruits, macerated in anisette	9
T,M
*Prunus avium* (L.) L.	cerezo; *fruit*: cereza, cereza silvestre	*Fruits*: raw	45	H-F 67 (1.18%)
Rosaceae; 227	*Liqueur*: fruits, macerated in anisette or liquor	19
T
*Digestive*^*:*^ fruits, liqueur in anisette or liquor	3
*Origanum vulgare* L.	orégano	*Seasoning*: flowering shoots, flavour pasta, stews, salads	27	H-F 66 (1.17%)
Lamiaceae; 44
*Sore throat, colds and phlegm*: flowering shoots, infusion with milk, *Malva sylvestris* or *Rosmarinus officinalis;* decoction, steam inhalations	14
T,PA
*Diarrhoea, stomach pain, digestive*: flowering shoots, infusion	11
*Relaxant*: flowering shoots, infusion	9
*Herbal tea*: flowering shoots, infusion	5
*Cydonia oblonga* Mill. †; Rosaceae; 542	membrillo	*Fruits:* jam; boiled with pears, apples, prunes and raisins	35	H-F 62 (1.10%)
*Depression* : fruits, liqueur in wine	11
*Liqueur*: fruits, macerated in wine	13
*Eyes inflammation*: fruits, decoction, washings	3
*Fragaria vesca* L.; 531	*plant and fruit*: amabia, amahueta, amayeta, fresa, mahueta, marrubia, mayeta, metra, fresa de monte, fresa pequeñita, fresa salvaje, fresa silvestre	*Fruits*: raw, boiled with sugar and wine	60	F 60 (1.06%)
T, M

⁎ *Patxaran: Prunus spinosa* fruits are macerated in anisette for five or six months with coffee beans, cinnamon bark and *Chamaemelum nobile* inflorescences.

⁎⁎ Pomada sanjuanera: Ointment prepared frying together many plants such as: *Achillea millefolium*, *Allium ampeloprasum*, *Anagallis arvensis*, *Calendula officinalis*, *Chamaemelum nobile*, *Cruciata glabra*, *Eleocharis quinqueflora*, *Glycyrrhiza glabra*, *Hedera helix*, *Hypericum hirsutum*, *Hypericum perforatum*, *Inula montana*, *Jasonia tuberosa*, *Lavandula latifolia*, *Malva sylvestris*, Mentha×piperita, *Pinus sylvestris*, *Plantago lanceolata*, *Plantago major*, *Potentilla erecta*, *Potentilla reptans*, Primula veris, *Pulmonaria longifolia*, *Ranunculus ficaria*, Rumex sp., *Sambucus nigra*, *Santolina chamaecyparissus*, *Symphytum tuberosum*, *Thymus vulgaris*, *Tussilago farfara*, *Verbascum thapsus* and *Verbena officinalis*.

⁎⁎⁎ Olives in brine: olives are macerated in water with salt until the bitter flavour has left. Then they are macerated in cold water, garlic, thyme and rosemary for two months

a Unless indicated by a cross (†) all species are non-cultivated; species which are both cultivated and non cultivated are indicated with two crosses (††).

b Collector: Rocío Alarcón.

c Comparison with other authors: T (Tardío et al., 2006), M (Menendez-Baceta et al., 2012), PA (Puente Amestoy, c. 1960).

d Use-category: H-F (health-food), F (food), M (medicine).
